# From perceptual rule transformation to listener attribution judgments: a blind-listening experiment on AI-generated, human–AI collaborative, and human-composed music

**DOI:** 10.3389/fpsyg.2026.1903971

**Published:** 2026-08-26

**Authors:** Junsong Chang, Yueqi Jing

**Affiliations:** 1 School of Music, Xinxiang University, Xinxiang, China; 2 School of Music, Henan University, Kaifeng, China; 3 School of Korean Music Theory, Chung-Ang University, Dongjak-gu, Republic of Korea

**Keywords:** aesthetic evaluation, AI music, blind listening, creator attribution judgment, endogenous perceptual bias, human–AI collaboration

## Abstract

This study examines listeners’ creator attribution judgments and aesthetic evaluations of AI-generated, human–AI collaborative, and human-composed music under blind-listening conditions. Within a framework of personal compositional style modeling, compositional experience was transformed into executable sampling constraints through natural-language interaction. This process is conceptualized in the present study as “perceptual rule transformation” and was used to generate 18 melodic excerpts. Seventy-one participants with music training completed tasks involving creator attribution judgment, attribution confidence rating, and aesthetic evaluation. The results partially supported H1: a statistically significant but very small association was observed between the actual compositional condition and listeners’ attribution judgments, χ^2^(4) = 23.076, *p* < 0.001, Cramér’s V = 0.095. Attribution judgments in the AI-generated and human–AI collaborative conditions were close to a random distribution, and even in the human-composed condition the majority of excerpts (54.7%) were misattributed. Thus, musically trained listeners could not reliably identify the compositional source of the excerpts, with only a modest attribution advantage for human-composed music. Significant differences in aesthetic evaluation were also found across the three compositional conditions. After controlling for actual compositional condition, attribution confidence, and individual participant differences, creator attribution judgment remained independently associated with aesthetic evaluation, and this association held even within the AI-generated condition, where the actual source of all excerpts was constant. These findings suggest that, in the absence of external authorship labels, listeners spontaneously form judgments about the creative agent of music. Such judgments are stably associated with aesthetic evaluation and may constitute an endogenous perceptual bias in the reception of AI-generated music.

## 1 Introduction

Artificial intelligence (AI) music technology has gradually shifted from rule-based and probabilistic algorithmic models to large-scale generative paradigms centered on machine learning (ML) and deep learning (DL), demonstrating increasingly powerful generative capacities across multiple musical tasks, including melody generation, polyphonic writing, and accompaniment generation (Briot et al., 2020). Existing scholarship on AI music has primarily focused on technical implementation, the evaluation of AI-generated creative outputs, creative agency, and ethical and legal issues. Comparatively less attention has been paid to composers’ concrete experiences of using AI systems and the practical logic through which such systems are incorporated into compositional processes (Newman et al., 2023; Ghvinjilia, 2025; Gioti, 2020).

A review by Newman et al. (2023) further indicates that, even within the field of music information retrieval (ISMIR), fewer than 20 studies over the past decade have directly examined AI-based music creation tools, and less than half of these studies sufficiently incorporated the perspectives of music creators during the tool-development stage. Even when creator perspectives were included, these studies tended to focus on professional users who had already adopted AI tools and were largely situated within Western musical traditions (Newman et al., 2023). Meanwhile, existing case studies have shown that customized generative models trained on an individual creator’s own materials can produce musical content that combines recognizable personal stylistic traits with a certain degree of novelty. However, such studies have not yet extended to the level of aesthetic evaluation, nor have they provided a more systematic empirical analysis of the creative practices involved in personalized model construction (Weaver, 2026). In addition, existing research has not yet examined works generated by personal style models constructed through natural-language interaction.

These gaps lead to the central question of the present study: from a practice-based perspective, how can a composer without professional programming expertise use natural language to transmit compositional ideas and experiential knowledge to an AI system through sustained interaction, thereby guiding the system to transform such knowledge into executable generative rules? Current literature remains limited in this respect, particularly in terms of systematic empirical investigation of this process. This study seeks to address this gap by providing a reference case for composers who possess advanced musical expertise but lack specialized computational training, showing how they may construct personalized AI-assisted compositional tools.

On this basis, the present study combines compositional practice with empirical research. A two-level collaborative creation framework was established. First, the author selected the main-melody MIDI files from sixteen original vocal works as the foundational corpus and gradually constructed a personal-style melody generator through sustained natural-language interaction with OpenAI Codex. The generator adopts a hybrid architecture: a small Transformer model produces probabilistic melodic distributions, while a rule-based layer constrains and filters the sampled outputs according to musical conditions such as cadence, rhythm, and tonality. The composer then provides continuous auditory feedback, which drives the iterative optimization of system rules and parameters.

Second, using this generator, the author conducted compositional practice under three conditions reflecting different degrees of human involvement: AI-generated music, in which the generator output was used directly without post-processing; human–AI collaborative composition, in which the generator output was revised by the composer; and human-composed music, in which the composer created the material independently. These three compositional conditions constitute the experimental basis of the present study.

This study evaluates the outputs of the generator through a listening experiment from two perceptual dimensions: creator attribution judgment and aesthetic evaluation. An experimental method was adopted to examine whether listeners could make creator attribution judgments and aesthetic evaluations of works produced under the three compositional conditions. This empirical question cannot be answered through technical analysis alone. In this study, creator attribution judgment is not used as an ontological claim in the philosophical sense. Rather, it is defined as a task-specific concept within the listening experiment, referring to listeners’ judgments during the listening process regarding whether a musical excerpt is more likely to have been created by a human composer, generated by AI, or produced through human–AI collaboration.

Stammer et al. (2025) showed that composer identity information, perceived humanness, and individual listener factors can all influence listeners’ aesthetic evaluations of AI-generated music. However, existing research has not sufficiently examined whether creator attribution judgments formed spontaneously under blind-listening conditions, without any source labels, are associated with aesthetic evaluation. Building on this gap, the present study treats creator attribution judgment and aesthetic evaluation as two perceptual validation dimensions. It investigates the relationship between creator attribution judgment and aesthetic evaluation under blind-listening conditions and further assesses the perceptual reception of materials generated by a personal-style model.

The significance of this study lies in both theoretical and methodological contributions. Theoretically, it shifts the focus from the evaluation of AI-generated musical outputs to the process through which a generator is constructed through natural-language interaction, thereby supplementing the relatively underdeveloped creator-practice dimension in AI music research. Methodologically, it proposes an integrated research design that proceeds from the organization of personal-style materials and the interactive construction of a generator to the perceptual validation of generative outcomes through a listening experiment. This design offers an analyzable and transferable practice-based model for constructing personal-style compositional systems in the age of AI. Empirically, the study further examines whether listeners’ subjective attribution judgments, formed in the absence of external authorship labels, are independently associated with aesthetic evaluation, thereby linking personal style modeling with the psychological mechanisms underlying the reception of AI music.

## 2 Literature review and research hypotheses

### 2.1 Technical positioning of the study

AI composition can be understood as an extension of algorithmic composition in the era of deep learning. Its defining feature lies in the model’s capacity to autonomously extract generative patterns from data, rather than relying primarily on manually predefined rules (Briot and Pachet, 2020; Fernández and Vico, 2013). Building on this technological trajectory, the personal compositional style model developed in the present study introduces natural language as a mediating mechanism through which the composer’s experiential knowledge is transformed into executable generative rules.

From a technical-genealogical perspective, the present study is directly related to two lines of development. The first is the trajectory of style learning. Cope’s EMI system (Cope, 1989, 1992, 1996) shifted the focus of generation from rule-based operations to the extraction of stylistic features, providing an early example of style modeling based on an individual corpus. The second is the trajectory of interactivity. Pachet’s Continuator (Pachet, 2003) was among the first systems to realize real-time responses to performer input, anticipating the shift of AI music systems from automatic generation toward interactive construction. The introduction of natural language further lowers the threshold of interaction, enabling creators without programming backgrounds to participate in the construction of generative rules through linguistic description (Zhao et al., 2025).

The concept of “perceptual rule transformation” proposed in this study should be distinguished from three adjacent notions. It differs from prompt engineering in its object and product: prompt engineering optimizes natural-language inputs to steer the outputs of a pre-trained generative model on a per-request basis, whereas perceptual rule transformation uses natural language to externalize a composer’s tacit perceptual criteria—melodic fluency, vocality, breathing points, cadential strength—into persistent, inspectable sampling constraints, filtering conditions, and parameter rules that become part of the system itself. It is related to, but narrower than, interactive machine learning and human-in-the-loop generation, which describe general system architectures in which human feedback iteratively shapes model behavior. Perceptual rule transformation designates the specific translational step within such a loop: the conversion of embodied, perceptually grounded compositional judgment into executable symbolic rules via natural-language description. In this sense, the concept characterizes a knowledge-transformation process on the composer’s side rather than a system architecture on the technical side, and it is this process—rather than the loop structure as such—that the present study documents and conceptualizes.

### 2.2 Research on creator attribution judgment and aesthetic evaluation

With the development of AI music research, increasing scholarly attention has been directed toward the evaluation of generated music. Chu et al. (2022) investigated human satisfaction with deep-learning-based automatic symbolic music generation models. They proposed several subjective evaluation indicators and examined how different models and representational strategies influenced listener satisfaction. Their findings suggest that the evaluation of AI-generated music cannot rely solely on technical metrics; rather, it must also consider listeners’ subjective perceptions of melodicity, rhythmicity, structural coherence, and overall acceptance. This study provides a methodological reference for incorporating perceived musical quality into the aesthetic evaluation framework of the present research (Chu et al., 2022).

Di Dio et al. (2025) examined aesthetic judgments of visual artworks under different authorship-label conditions. They distinguished aesthetic evaluation into two dimensions, namely beauty judgment and liking judgment, and compared evaluation results between conditions with no authorship information and conditions with explicit authorship cues. Their findings indicate that aesthetic evaluation is not determined solely by the formal properties of the artwork itself; authorship information can significantly intervene in the evaluative process. This study is relevant to the present research because it suggests that aesthetic evaluation can be divided at least into liking, which is more closely related to affective response, and beauty, which is more closely related to value judgment (Di Dio et al., 2025).

Bellaiche et al. (2023) further found that even AI-generated artworks received higher evaluations in dimensions such as liking, beauty, profundity, and value when they were labeled as human-created. This indicates a general negative bias toward AI-created artworks compared with works labeled as human-created (Bellaiche et al., 2023). However, the focus of their study remained on the effect of external label information on evaluation, rather than on audiences’ spontaneously formed creator attribution judgments under blind-evaluation conditions.

Shank et al. (2023) used musical excerpts in different styles to examine listeners’ liking ratings and their judgments of creator attribution. The results showed that participants were more likely to attribute electronic music to AI creation, and that music they believed to be AI-created received lower liking ratings (Shank et al., 2023). This study is closely related to the present research because it already connects creator attribution judgment with liking. Nevertheless, its main framework remains a binary distinction between human and AI creation and does not incorporate human–AI collaborative creation as an intermediate condition.

Similarly, Tubadji et al. (2021) discussed why people tend to overestimate the value of human-created music from the perspective of AI music creation and human emotional value (Tubadji et al., 2021). Ansani et al. (2025) extended the focus from composer identity to performer identity. Their findings showed that, even when the musical material was identical, listeners perceived human performance as better and more moving than AI performance, suggesting that identity information directly affects aesthetic experience (Ansani et al., 2025). Stammer et al. (2025) further demonstrated that creator identity information, personality traits, musical preferences, attitudes toward AI, and perceived anthropomorphism can all influence evaluations of AI-generated music (Stammer et al., 2025).

Taken together, existing studies have systematically examined the influence of creative-agent identity on the aesthetic reception of art and music from multiple perspectives, including the dimensional structure of aesthetic evaluation (Di Dio et al., 2025), creator identity information (Bellaiche et al., 2023; Shank et al., 2023; Tubadji et al., 2021), perceptual differences caused by performer identity (Ansani et al., 2025), and listeners’ pre-existing attitudes toward AI (Stammer et al., 2025). Three major lines of research can be identified. First, the evaluation of AI-generated music needs to be grounded in listeners’ subjective perception. Second, the aesthetic evaluation of artistic and musical works can be differentiated into multiple dimensions, such as liking, beauty, perceived quality, and emotional response. Third, authorship labels, performer-source information, and perceived humanness may all influence audiences’ aesthetic evaluations of artistic and musical works.

### 2.3 Endogenous perceptual bias

Most existing studies have examined the influence of identity information on aesthetic evaluation by explicitly providing participants with external labels, such as “human-created” or “AI-created.” In contrast, the present study focuses on creator attribution judgments that listeners form spontaneously under blind-listening conditions. In this study, the term “endogenous perceptual bias” refers to the phenomenon in which listeners, in the absence of external authorship labels, independently infer the creative agent of a musical work on the basis of the musical material itself, and where such attribution judgments show a stable association with aesthetic evaluation.

### 2.4 Research hypotheses

Despite the contributions of previous research, several limitations remain. First, many studies provide participants with information about the source of creation and then examine whether aesthetic evaluations change accordingly. This paradigm can reveal the effect of authorship labels on aesthetic evaluation, but it cannot explain whether listeners form creator attribution judgments based on the music itself when no external labels are provided, nor whether such judgments are associated with aesthetic evaluation. Second, most studies adopt a binary framework that contrasts human creation with AI creation, while paying less attention to human–AI collaborative creation as an intermediate condition.

Accordingly, the present study examines three compositional conditions: AI-generated music, human–AI collaborative composition, and human-composed music. Under blind-listening conditions, with no source labels provided, the study investigates listeners’ creator attribution judgments of musical works and further analyzes whether these judgments are systematically associated with aesthetic evaluation. In doing so, this study extends previous research by connecting interactive personal style modeling with the perceptual reception of AI music.

Based on the above discussion, the following hypotheses are proposed:

*H1:* Musical works produced under the three actual compositional conditions will elicit different distributions of creator attribution judgments. Specifically, under blind-listening conditions, AI-generated, human–AI collaborative, and human-composed works will differ in the proportions of listener attribution judgments.

*H2:* Within the range of materials used in this study, works produced under different actual compositional conditions will differ in aesthetic evaluation. Specifically, AI-generated, human–AI collaborative, and human-composed works will differ in overall aesthetic evaluation; the four content domains (liking, beauty, perceived musical quality, and emotional expressiveness) are examined as exploratory supplementary analyses.

H1 and H2 examine overall perceptual differences across the three compositional conditions. H3 further tests whether subjective creator attribution judgment is independently associated with aesthetic evaluation, which constitutes the core hypothesis of this study concerning endogenous perceptual bias.

*H3:* After controlling for actual compositional condition, attribution confidence, and individual participant differences, listeners’ creator attribution judgments will remain independently associated with aesthetic evaluation. Specifically, compared with works attributed to AI generation, works attributed to human composition are expected to receive higher aesthetic ratings.

## 3 Construction of a personal compositional style model combining neural network generation and rule-based constraints

### 3.1 Research purpose and corpus selection

In this study, “personal compositional style” refers primarily to the relatively stable melodic features that can be represented through MIDI-based symbolic encoding in the author’s existing vocal melodies. These features include pitch organization, rhythmic tendencies, phrase length, cadential patterns, melodic direction, and vocal-line treatment. More complex dimensions, such as orchestration, harmonic arrangement, and large-scale formal structure, are not included in the scope of the present model. As discussed above, existing research on AI music has paid limited attention to how composers construct personal style models based on their own materials through natural-language interaction. This section addresses that issue by describing the construction of the system used to generate the experimental stimuli in this study.

The present study selected the main-melody MIDI files from sixteen existing vocal works by the author as the foundational corpus. The use of a personal corpus was motivated by two considerations. First, the author could evaluate, on the basis of his own compositional experience, whether the generated outputs approximated his original melodic language. Second, monophonic melodic materials are suitable for modeling dimensions such as pitch, rhythm, phrase structure, cadence, and vocality. The system was not designed to automatically complete full songs. Rather, its aim was to generate 16-bar melodies with phrase-level coherence, sectional organization, and vocal-line characteristics under small-sample conditions.

During system construction, the composer provided the training corpus and, under the author’s direction, used OpenAI Codex to implement the code and iteratively refine the system. Based on auditory evaluation, the composer continuously proposed modification requirements. Experiential judgments concerning melodic fluency, vocality, breathing points, cadential strength, tonal stability, and sectional relationships were translated through natural language into executable sampling constraints, filtering conditions, and parameter-adjustment rules. This process is conceptualized in the present study as “perceptual rule transformation”.

### 3.2 MIDI data processing and melodic tokenization

This study used MIDI data for model training. The original MIDI files were exported from Sibelius and may have contained a small number of overlapping notes, abnormal durations, ornamental notes, and other factors that could interfere with training. Therefore, the first step in system construction was data inspection and cleaning. With the assistance of Codex, the system identified the track structure of each MIDI file, confirmed that the main melody was concentrated in a single track, and normalized issues such as overlapping notes and abnormal durations. The cleaned melodic data were then exported for subsequent training.

Because the personal corpus was limited in size, directly using entire melodies as training units would have increased the risk that the model would memorize the original materials rather than develop effective generalization. To alleviate this problem, the melodies were segmented into 2-bar and 4-bar phrase units. This procedure increased the number of training samples and encouraged the model to prioritize the learning of local rhythmic patterns, intervallic connections, phrase endings, and intra-bar accent positions.

In terms of input representation, the system adopted a melodic tokenization scheme in which each note event was described by pitch, duration, metric position within the bar, and bar number. The metric-position information indicates the beat placement of each note within the bar. The small Transformer Decoder trained in this study functions as an autoregressive melodic token model. It predicts the probability distribution of the next token based on the preceding token sequence and generates new melodic sequences through continuous sampling. It should be emphasized that the neural network provides the main generative probability distribution, whereas the rule-based layer only guides, filters, and constrains the sampling process. It does not replace the generative function of the model itself.

### 3.3 Rule-constrained sampling and composer feedback iteration

In the initial generation stage, the model outputs already showed a certain degree of melodic coherence. However, several problems remained, including uneven distribution of rests, excessive density of short durations, loose melodic direction, insufficient sectional contrast, and weak cadential stability. To address these issues, a rule-constrained sampling mechanism was added on the basis of the probability distributions produced by the Transformer. Through probability biasing, candidate masking, and output filtering, the system guided the melodies toward greater suitability for vocal melodic writing in terms of melodic contour, high points, closure, and overall stability.

After the generation of 4-bar phrases became relatively stable, the system was further extended to 8-bar and 16-bar sections. This extension was not a simple lengthening of the melody. Instead, it controlled material relationships and variations between preceding and subsequent phrases so that the generated outputs could form a relatively clear sense of sectional organization. During sampling, the system incorporated constraints related to continuity, variation, cadential tones, and breathing positions, enabling the 16-bar melodies to achieve greater stability in structure, tonality, and vocality.

The system constructed in this study is not a closed automatic generation process. Rather, it is a collaborative mechanism involving the composer, the neural network model, and the natural-language programming tool. The composer first identified problems in the generated results through auditory judgment, such as insufficient melodic coherence, unnatural breathing points, unclear cadential effects, or inadequate sectional contrast. The composer then proposed modification requirements in natural language, and Codex translated these requirements into executable code rules and sampling parameters. In this way, the system formed a personal melodic style generation mechanism consisting of “small Transformer-based primary generation—rule-constrained sampling—composer auditory feedback iteration”.

Through the above process, the present study obtained the AI-generated materials used in the subsequent listening experiment. On this basis, human–AI collaborative materials and independently human-composed materials were further produced. The purpose of this section is not to demonstrate that the model outperforms other generative systems in technical performance. Rather, it clarifies how the experimental materials were produced within a personal style modeling framework and provides the methodological foundation for the later experimental analysis of creator attribution judgment and aesthetic evaluation.

## 4 Overall framework of the experimental design

### 4.1 Overall design

This study adopted a one-group design in which all participants rated all 18 excerpts. It should be noted that, although there was no between-participants manipulation, each excerpt was fixed to one compositional condition; compositional condition therefore varied between items (six items per condition) and was crossed with participants, rather than being manipulated within participants in the conventional sense. The same group of participants listened to 18 melodic excerpts under blind-listening conditions and completed creator attribution judgment, attribution confidence rating, and aesthetic evaluation after each excerpt. The three actual compositional conditions—AI-generated music, human–AI collaborative composition, and human-composed music—each included six stimulus materials, resulting in a total of 18 excerpts. These three categories corresponded, respectively, to direct outputs from the generator, composer-revised outputs after generation, and melodies independently composed by the composer.

### 4.2 Stimulus materials

The stimulus materials consisted of three groups: AI-generated, human–AI collaborative, and human-composed music. A total of 30 candidate excerpts were prepared. Among them, S1–S10 were human–AI collaborative compositions, S11–S20 were AI-generated compositions, and S21–S30 were human-composed compositions. The AI-generated group consisted of direct outputs from the personal-style melody generator without any post-processing. The human–AI collaborative group consisted of generator outputs that were locally revised by the composer. The revisions were limited to pitch and rhythm and did not alter the overall formal structure. A 20% ceiling on the proportion of revised notes was set *a priori* as an operational bound to ensure that revisions remained local refinements rather than re-composition; the specific value is an operational convention rather than a perceptually derived threshold, which is acknowledged as a limitation. Both the original and revised files have been retained and are available in the OSF repository (https://doi.org/10.17605/OSF.IO/ZUSJN). The human-composed group was independently created by the composer without using the generator and was matched as closely as possible with the AI-generated condition in terms of modal type, tempo range, and approximate register. For transparency, it should be stated explicitly that the composer referred to throughout this study is the first author, who also provided the training corpus, directed the construction of the generator, and produced the stimuli in all three conditions. The human-composed excerpts were pre-existing works written between 2020 and 2023, before the generator was constructed; at the time of composition, the author did not and could not know that these works would later serve as the human-composed condition in a perception experiment.

For the main experiment, six excerpts were selected from each candidate group, resulting in 18 melodic excerpts in total. Each excerpt lasted approximately 20–30 s. All materials were produced according to a unified audio-production standard, using the same virtual instrument, identical export parameters, loudness processing, and reverb settings, in order to minimize the potential influence of audio-production differences on the experimental results.

Before the main experiment, three experts with backgrounds in composition or music theory were invited to conduct a blind quality evaluation of the 30 candidate materials. The experts were unaware of the actual compositional source of each excerpt and rated the materials on a 7-point scale across five dimensions: melodic completeness, musical fluency, structural clarity, cadential sense, and overall musical quality. To examine the consistency of expert ratings, an agreement analysis was conducted using the mean score of the five dimensions assigned by each expert to the same material. The results showed that the absolute-agreement ICC for a single expert was ICC(A,l) = 0.727, and the absolute-agreement ICC for the mean ratings of the three experts was ICC(A,k) = 0.889, indicating good consistency in the averaged expert ratings. Kendall’s W was further calculated to assess the degree of agreement among the three experts in ranking the quality of the candidate materials. The results indicated a high level of agreement, Kendall’s W = 0.838, χ^2^(29) = 72.914, *p* < 0.001.

Based on these results, the mean ratings of the three experts were used as the basis for material selection. Within each compositional condition, excerpts with relatively high quality scores and without obvious structural or audio-production problems were selected for inclusion in the main experiment. The selected materials are presented in Table 1. The mean expert-rated quality scores of the three selected material groups were all at a relatively high level: 4.633 for the AI-generated group, 4.767 for the human–AI collaborative group, and 4.789 for the human-composed group. The overall mean score of the 18 selected excerpts was 4.730. A one-way analysis of variance using the selected excerpts as the unit of analysis showed no significant difference in expert-rated quality among the three material groups, F(2, 15) = 0.256, *p* = 0.777. It must be acknowledged, however, that a non-significant F test based on six items per group has very low power, and non-significance cannot be taken as evidence of equivalence. This analysis is therefore reported only as a screening check indicating the absence of gross quality disparities among the selected materials, not as a demonstration that the three groups were equivalent in quality. Residual quality differences remain a competing explanation for condition effects and are addressed in the Limitations section and in the within-condition analyses (Section 5.7.4).

**Table 1:** Stimulus materials and expert screening results.

Compositional condition	Candidate range	Selected candidate material IDs	Mean expert quality score	Preparation method
AI-generated	S11-S20	S20, S13, S16, S11, S14, S12	4.633	Direct output from the generator without post-processing
Human-AI collaborative composition	S1-S10	S6, S9, S7, S10, S1, S8	4.767	Generator output locally revised by the composer
Human-composed	S21-S30	S22, S24, S25, S30, S21, S23	4.789	Completely independently composed by the composer

Candidate material IDs are ordered from high to low according to the mean expert quality score. The mean expert quality score is the average for the six selected excerpts based on five rating dimensions and the ratings of three experts. Six excerpts were selected for each group, and each excerpt lasted approximately 20–30 s.

The purpose of expert screening was not to demonstrate that the three types of materials were completely identical in quality, but rather to reduce, as far as possible before the main listening experiment, the potential interference of obviously low-quality materials and quality differences with the results of creator attribution judgment and aesthetic evaluation. In the main experiment, the selected materials were renumbered and presented in randomized order. The candidate numbers shown in the table were used only to document the material sources during the expert-screening stage and do not represent the presentation order in the main experiment. All 18 stimulus audio files used in the main experiment, covering the three compositional conditions, are openly available in the Open Science Framework repository (https://doi.org/10.17605/OSF.IO/ZUSJN).

### 4.3 Participants

This study was designed to recruit approximately 100 music-major students as formal participants. All participants were required to have systematic training in music theory and experience in aural skills or listening-based musical judgment. The inclusion criteria were as follows: (1) undergraduate music majors in their third year or above; (2) no self-reported hearing impairment; and (3) no prior exposure to the stimulus materials used in this study.

During formal data screening, the exclusion criteria were predefined as follows: (1) failure to complete the listening tasks for all 18 excerpts; (2) obviously abnormal response time; (3) clear patterned or mechanical responding; and (4) missing values in key variables. Any sample meeting one or more of these criteria was excluded from subsequent statistical analyses. In practice, criteria (1)–(4) were jointly operationalized through a single completion-time threshold, as detailed in Section 5.1.

Before the experiment, participants were informed that the study aimed to examine listeners’ perception and evaluation of different melodic excerpts. However, the specific compositional condition corresponding to each stimulus was not disclosed. All participants were required to provide informed consent. After the experiment, the researchers conducted a debriefing session, in which participants were informed of the study purpose, the three compositional conditions, and the rationale for the experimental design.

The study aimed to obtain no fewer than 70 valid samples. The sample-size estimation was primarily based on the one-way repeated-measures analysis of variance involved in H2. An *a priori* power analysis was conducted using G*Power. Under the conditions of a medium effect size of *f* = 0.25, a significance level of *α* = 0.05, and statistical power of 1 − *β* = 0.80, the minimum required sample size for a one-way repeated-measures ANOVA was approximately 52 participants. Therefore, no fewer than 70 valid participants would meet the sample-size requirement for the repeated-measures analysis involved in H2. Because G*Power does not accommodate the trial-level mixed-effects model used for H3, a separate Monte Carlo simulation-based sensitivity power analysis, implemented in Python, was conducted for the attribution effect under the realized design (71 participants × 18 trials), using the variance components estimated from the data. Statistical power reached 75.6% for an attribution effect of B = 0.15, 94.4% for B = 0.20, and effectively 100% for B ≥ 0.30. The attribution effects actually observed (|B| = 0.81–0.93; Section 5.7) far exceed the smallest effect detectable with 80% power, indicating that the design provided ample power for the H3 test.

### 4.4 Experimental procedure

The experiment was conducted offline using a standardized procedure. Each participant first read and signed the informed consent form. They then completed the listening task using headphones or in a quiet listening environment. Before the formal task, one to two practice excerpts were presented to familiarize participants with the question format and rating procedure. The practice excerpts were not included in the formal data analysis.

During the formal experiment, the 18 melodic excerpts were presented in randomized order. After each excerpt, participants completed the following items in sequence: (1) creator attribution judgment; (2) attribution confidence rating; and (3) aesthetic evaluation scale. To reduce order effects, the presentation order of the materials was randomized across participants.

### 4.5 Measures

Creator attribution judgment was measured using a three-option forced-choice item, accompanied by a 7-point attribution confidence scale ranging from 1 = completely uncertain to 7 = highly certain. The aesthetic evaluation scale consisted of eight items rated on a 7-point Likert scale, ranging from 1 = strongly disagree to 7 = strongly agree. The eight items were drawn from four content domains identified in prior research: liking, beauty, perceived musical quality, and emotional expressiveness. As reported in Section 5.4, a principal component analysis indicated that the eight items formed a single factor; the overall aesthetic evaluation score was therefore used as the outcome variable, and domain-level scores are presented for exploratory purposes only. The full measurement items are presented in Table 2.

**Table 2:** Content domains and items of the aesthetic evaluation scale.

Content domain	No. of items	Reference sources	Items
Liking	2 items	Shank et al. (2023) and Ansani et al. (2025)	I like this piece of music I would like to listen to this piece of music again.
Beauty	2 items	Di Dio et al. (2025) and Bellaiche et al. (2023)	This piece of music has aesthetic beauty This piece of music is artistically impressive.
Perceived musical quality	2 items	Chu et al. (2022) and Ansani et al. (2025)	This piece of music is of high overall quality This melody is fluent and logical.
Emotional expressiveness	2 items	Ansani et al. (2025)	This piece of music conveys strong emotion I experienced emotional resonance while listening to this piece of music.

For four content domains of aesthetic evaluation: liking, beauty, perceived musical quality, and emotional expressiveness. The mean of these four domain scores was further computed to form an overall aesthetic evaluation score. The overall aesthetic evaluation score was used as the primary outcome variable for the main analysis of H2, which examined differences in aesthetic evaluation across the three actual compositional conditions. Scores for the four content domains were reported as exploratory supplementary results only, given the single-factor structure of the scale.

For H3, this study employed a trial-level linear mixed-effects model rather than a simple correlation analysis based only on the 18 excerpts. Specifically, each single rating record provided by each participant for each stimulus was treated as the unit of analysis. After controlling for actual compositional condition and attribution confidence, participant ID was included as a random intercept to account for individual differences in overall rating tendencies. Attribution confidence was included as a covariate in the primary model in order to examine whether the attribution–evaluation association held beyond listeners’ general decisiveness in the task. It is acknowledged, however, that confidence is plausibly downstream of the same perceptual process that produces both the attribution and the aesthetic rating; if so, conditioning on it constitutes adjustment for a potential mediator rather than a neutral control, which can bias the attribution coefficients. Because the causal status of confidence cannot be determined from the present design, the model was estimated both with and without the confidence covariate, and the sensitivity of the attribution effect to this decision is reported in Section 5.7.3. Because the number of stimulus materials was limited (18) and each stimulus belonged to a specific actual compositional condition, the primary model included no random intercept for stimulus material. It should be made explicit that omitting stimulus-level random effects in a design with repeated ratings of the same 18 items is a known route to inflated Type I error, because the model treats the 1,278 observations as carrying more independent information than they do; the reported *p* values should be read with this in mind. To address this directly, a crossed random-effects model with random intercepts for both participant and stimulus was estimated as a robustness check and is reported in Section 5.7.5.

Given that each aesthetic evaluation measure contained only two items, the reliability analysis reported Cronbach’s *α* coefficients and also referred to inter-item correlations to assist in evaluating the internal consistency of each dimension. Before formal hypothesis testing, the internal consistency of the aesthetic evaluation scale was first examined. Subsequent statistical analyses were conducted only after the reliability reached an acceptable level.

### 4.6 Data analysis

All statistical analyses were conducted in IBM SPSS Statistics 27, except for the simulation-based power analysis, which was implemented in Python. Mixed-effects models were estimated with restricted maximum likelihood (REML), with degrees of freedom approximated by the Satterthwaite method, which accounts for the non-integer degrees of freedom reported throughout.

The expert pretest was used primarily as a procedure for stimulus selection and quality control and was not included in the formal hypothesis testing. The formal data analysis focused on the listening data from valid participants and consisted of four parts: reliability analysis, H1 testing, H2 testing, and H3 testing.

First, the reliability of the aesthetic evaluation scale was examined. Cronbach’s *α* coefficients were calculated to evaluate the internal consistency of the four content domains—as well as the overall scale. In addition, inter-item correlations were used as supplementary indicators of internal consistency.

For H1, a confusion matrix and chi-square test were used to analyze the relationship between actual compositional condition and creator attribution judgment. H1 tested differences in the distribution of attribution judgments rather than the accuracy of source identification. If the distributions of attribution judgments differed significantly across the three actual compositional conditions, this would indicate that materials from different sources elicited different attribution tendencies. It should be emphasized that such a result does not necessarily mean that listeners could accurately identify the actual source of the materials. Therefore, the specific proportions of the three attribution categories within each condition were also reported to describe listeners’ attribution tendencies. In addition, a one-way within-participants analysis of variance was conducted with attribution confidence as the dependent variable as a supplementary analysis.

For H2, a one-way repeated-measures analysis of variance was conducted to examine differences in aesthetic evaluation across the three actual compositional conditions. Actual compositional condition was entered as the within-participants independent variable, and the overall aesthetic evaluation score was used as the dependent variable. If the main effect was significant, Bonferroni-corrected *post hoc* comparisons were performed. The four content domains of liking, beauty, perceived musical quality, and emotional expressiveness were reported separately as exploratory supplementary analyses.

For H3, a linear mixed-effects model was used, with each single rating record formed by each participant for each stimulus material treated as the unit of analysis. The overall aesthetic evaluation score was entered as the dependent variable, creator attribution judgment was entered as the core independent variable, and actual compositional condition and attribution confidence were included as control variables. Participant ID was included as a random intercept to control for individual differences in overall rating tendencies.

Because this study used 18 stimulus excerpts and each excerpt was fixed within a particular actual compositional condition, material-level differences were structurally coupled with actual compositional condition to some extent. Considering model stability and interpretive clarity, this study mainly reports the linear mixed-effects model including participant-level random intercepts. The limitation concerning the control of stimulus-material-level differences is addressed in the discussion section. If creator attribution judgment remains independently associated with aesthetic evaluation after controlling for actual compositional condition, attribution confidence, and individual participant differences—especially if trials attributed to “human composition” receive higher ratings than trials attributed to “AI generation”—this would indicate an independent association between listeners’ spontaneously formed creator attribution judgments and aesthetic evaluation.

### 4.7 Informed consent procedure

Before data collection, this study implemented a standardized written informed consent procedure for all participants. Prior to completing the experimental task, each participant received an informed consent form written in clear and accessible language. The form explained the purpose and background of the study, the experimental procedure and estimated duration, the entirely voluntary nature of participation, and participants’ right to withdraw at any stage without any negative consequences. The form also clearly stated that the study would not collect any personally identifiable information and that all data would be used solely for academic research purposes. Only participants who fully understood the above information and signed the informed consent form were allowed to proceed to the formal experiment. This procedure was designed to protect participants’ autonomy and right to be informed and complied with the basic ethical norms of informed consent in social science research.

### 4.8 Anonymization and data access control

To protect participant privacy, this study strictly implemented an anonymization protocol throughout data collection and analysis. The study did not collect names, contact information, identification numbers, or any other personally identifiable information. Each questionnaire and experimental record was assigned a unique numerical code, and no association existed between this code and the participant’s personal identity. All data were stored in encrypted form on access-controlled digital devices and were accessible only to the principal researcher and authorized members of the research team. Data analysis was conducted strictly at the aggregate level, and no individual-level data were presented in any research report. The experimental dataset (including stimulus audio files and analysis code) has been publicly stored on the OSF platform (https://doi.org/10.17605/OSF.IO/ZUSJN). These measures ensured participant privacy and data security throughout the research process.

### 4.9 Ethical statement

From the perspective of research design, this study was a non-interventional listening experiment that used standardized stimulus materials and self-report scales to collect participants’ perceptual and evaluative data. The study did not involve substantive behavioral intervention, health risks, or meaningful alteration of participants’ daily life conditions. All measured variables reflected participants’ subjective perception and aesthetic judgment of musical materials during the listening task.

The experiment adopted a blind-listening procedure. Before participants completed the creator attribution judgment and aesthetic evaluation tasks, they were not informed of the actual compositional source of the stimulus materials. This procedure was a necessary component of the experimental design and was not intended as deception. To protect participants’ rights, all participants were debriefed after the experiment. During the debriefing, the researchers explained the purpose of the study, the rationale of the experimental design, and the actual compositional conditions of the stimulus materials, and participants were given the opportunity to ask questions. After receiving the complete research information, participants retained the right to withdraw their data.

This study was reviewed and approved by the Ethics Review Committee of Xinxiang University, China, under approval number XYKJLL-2026031. All experimental procedures were implemented only after ethical approval had been obtained.

## 5 Results

### 5.1 Sample screening and valid sample description

This study originally planned to recruit 100 music-major students to participate in the experiment, and 100 questionnaires were actually collected. After data screening, 29 responses (29.0%) were excluded. Screening was operationalized through a single pre-specified criterion: total completion time. Because full playback of the 18 excerpts (approximately 20–30 s each) plus responding requires a minimum realistic duration, responses completed in less than approximately 7.4 min were judged insufficient for genuine listening and evaluation of all 18 excerpts and were classified as invalid. All 29 exclusions resulted from this response-time criterion; no responses were excluded on other grounds. The relatively high exclusion rate reflects the strictness of this duration criterion, which was applied to protect data quality. The final valid sample consisted of 71 participants, yielding a valid response rate of 71.0%.

All valid participants were third-year or higher undergraduate students majoring in music and had received systematic training in music theory and aural skills. The data collection focused primarily on the inclusion criteria relevant to the present study, including musical training background, completion of all listening tasks, and absence of prior exposure to the experimental materials. Demographic variables such as gender and academic year were not included in the main analytical models. This limitation is further addressed in the limitations section.

Based on the final sample of 71 valid participants and 18 stimulus excerpts, a total of 1,278 valid single-trial rating records were obtained. These records were used in the subsequent analyses of creator attribution judgment, attribution confidence, and aesthetic evaluation.

### 5.2 Expert screening and balance of stimulus materials

As described in Section 4.2, before the formal experiment, 18 formal stimulus materials were selected from 30 candidate excerpts through blind expert quality evaluation. The selected materials and expert-screening results are reported in Table 1. To avoid repeating the same expert pretest data in both the Methods and Results sections, this section only clarifies the function of this procedure for the subsequent analyses. The expert pretest was used primarily for stimulus selection and quality control and was not treated as a formal hypothesis test for H1, H2, or H3.

The main purpose of this screening procedure was to improve the comparability of the three types of materials in the formal listening experiment, rather than to statistically prove that the three groups of materials were completely equivalent in quality. After expert screening, the 18 excerpts included in the formal experiment were kept as balanced as possible in terms of duration, modal type, tempo range, register, rhythmic density, and overall musical quality. Accordingly, the subsequent analyses of creator attribution judgment, attribution confidence, and aesthetic evaluation could reduce, to some extent, the potential confounding influence of material-quality differences. Nevertheless, because the three groups of materials were produced through different creative processes, residual quality differences that could not be fully eliminated may still remain.

### 5.3 Descriptive statistics

Before conducting inferential statistical analyses, this section presents the descriptive statistics for the main variables, including the overall distribution of creator attribution judgments, the means and standard deviations of each aesthetic evaluation measure under the three actual compositional conditions, and the basic pattern of attribution confidence. These results provide an overall view of the data structure before formal hypothesis testing.

#### 5.3.1 Overall distribution of creator attribution judgments

A total of 1,278 valid attribution judgments were collected in this study, corresponding to 71 participants × 18 stimulus excerpts. The specific distribution of listeners’ attribution judgments under each actual compositional condition is presented in Table 3. Overall, the proportions of the three attribution categories were relatively close, with a slight tendency toward human-composed attribution at the overall level (36.2%), although the magnitude of this deviation was limited.

**Table 3:** Distribution of creator attribution judgments under different actual compositional conditions (*N* = 1,278).

Attribution judgment category	AI-generated condition *n* (%)	Human-AI collaborative condition *n* (%)	Human-composed condition *n* (%)	Total *n* (%)
Attributed to AI generation	133 (31.2%)	138 (32.4%)	106 (24.9%)	377 (29.5%)
Attributed to human-AI collaboration	155 (36.4%)	156 (36.6%)	127 (29.8%)	438 (34.3%)
Attributed to human composition	138 (32.4%)	132 (31.0%)	193 (45.3%)	463 (36.2%)
Total	426 (100%)	426 (100%)	426 (100%)	1,278 (100%)

Values in parentheses are percentages within each actual compositional condition and overall proportions in the total column.

When examined by condition, attribution judgments for the AI-generated and human–AI collaborative conditions were both close to a uniform distribution, with differences among the three attribution categories not exceeding five percentage points. In contrast, under the human-composed condition, the proportion of excerpts attributed to human composition reached 45.3%, which was clearly higher than the other two attribution categories. This pattern indicates a relative attribution advantage for human-composed materials.

#### 5.3.2 Descriptive statistics of aesthetic evaluation across compositional conditions

The means and standard deviations of the overall aesthetic evaluation score and the four content domains across the three actual compositional conditions are presented in Table 4. For the overall score, the three conditions showed a consistent graded pattern. The human-composed condition received the highest mean score (M = 4.252, SD = 1.002), followed by the human–AI collaborative condition (M = 4.007, SD = 0.955), while the AI-generated condition received the lowest mean score (M = 3.823, SD = 0.981).

**Table 4:** Descriptive statistics of aesthetic evaluation dimensions across actual compositional conditions (*N* = 71).

Content domain	AI-generated M (SD)	Human-AI collaborative M (SD)	Human-composed M (SD)	Overall M (SD)	Scale range
Overall aesthetic evaluation	3.823 (0.981)	4.007 (0.955)	4.252 (1.002)	4.027 (0.990)	1–7
Liking	3.843 (1.028)	4.045 (0.990)	4.295 (1.053)	4.061 (1.024)	1–7
Beauty	3.805 (1.004)	3.975 (0.957)	4.230 (1.019)	4.003 (0.993)	1–7
Perceived musical quality	3.930 (0.978)	4.088 (0.952)	4.354 (1.012)	4.124 (0.981)	1–7
Emotional expressiveness	3.716 (1.038)	3.919 (1.055)	4.127 (1.041)	3.921 (1.045)	1–7

*N* = 71 at the participant level; each participant’s score was averaged across six excerpts. The overall aesthetic evaluation score was computed as the mean of the four content domains. A 7-point Likert scale was used, with higher scores indicating more positive evaluations.

The descriptive results for the four content domains were fully consistent with the pattern observed in the overall score, showing the same trend of human-composed > human–AI collaborative > AI-generated. The standard deviations across conditions ranged from 0.95 to 1.06, indicating a comparable degree of within-group dispersion across the three compositional conditions.

#### 5.3.3 Descriptive statistics of attribution confidence

The descriptive statistics for attribution confidence across the different actual compositional conditions are presented in Table 5. The mean confidence scores in all three conditions were above the midpoint of the scale (4 points), with an overall mean of 4.606. This indicates that listeners generally showed a certain degree of subjective confidence when making creator attribution judgments.

**Table 5:** Descriptive statistics of attribution confidence across actual compositional conditions (*N* = 71).

Compositional condition	M	SD	Median	Interpretation
AI-generated	4.524	0.976	4.500	Close to the scale midpoint; moderate confidence
Human-AI collaborative composition	4.540	0.882	4.500	Only 0.016 higher than the AI-generated condition
Human-composed	4.754	1.025	4.667	Highest among the three conditions; relatively confident
Overall	4.606	0.911	4.556	Overall above the scale midpoint (4 points)

*N* = 71. Confidence was measured on a 7-point scale (1 = completely uncertain, 7 = highly certain). Condition means indicate participants’ average confidence across six excerpts.

Among the three conditions, the mean confidence scores for the AI-generated condition (M = 4.524) and the human–AI collaborative condition (M = 4.540) were highly similar, with a difference of only 0.016. In contrast, the human-composed condition received a clearly higher mean confidence score (M = 4.754) than the other two conditions. This pattern preliminarily suggests that listeners had greater subjective confidence when judging materials produced under the human-composed condition.

### 5.4 Reliability and dimensionality of the aesthetic evaluation scale

Cronbach’s *α* coefficients were calculated to examine the reliability of the aesthetic evaluation scale. The results are presented in Table 6. The *α* coefficients for the four content domains were all above 0.95, and the inter-item correlations ranged from 0.919 to 0.952, indicating high internal consistency within each dimension. When all eight items were included in the analysis, the overall scale showed a Cronbach’s α of 0.987. The corrected item–total correlations ranged from 0.896 to 0.970, and deleting any single item did not produce an α value higher than the current level.

**Table 6:** Reliability analysis of the aesthetic evaluation scale (*N* = 71).

Content domain	Items	Cronbach’s *α*	Inter-item correlation r	Reliability evaluation
Liking	Q1, Q2	0.971	0.943	High
Beauty	Q3, Q4	0.960	0.926	High
Perceived musical quality	Q5, Q6	0.975	0.952	High
Emotional expressiveness	Q7, Q8	0.956	0.919	High
Overall scale (Q1-Q8)	8 items	0.987	0.896–0.970*	High

* In the overall scale row, the “inter-item correlation” column reports the range of corrected item-total correlations (0.896–0.970). Each of the four content domains contains two items. High α values are used as evidence of internal consistency and do not directly indicate structural validity.

These results indicate that the overall aesthetic evaluation score (Aes), constructed by averaging the four domain scores, has an acceptable reliability basis and can be used in the subsequent hypothesis testing. However, because each domain contains only two items and the semantic content of the paired items is relatively similar, the high α coefficients may partly reflect the strong thematic focus of the items. Therefore, the present study does not interpret the high reliability coefficients as direct evidence of sufficient structural validity. Inter-item correlations are also reported to assist in evaluating the internal consistency of each dimension and to avoid relying solely on Cronbach’s *α* when drawing conclusions.

To examine the dimensionality of the scale directly, a principal component analysis was conducted on the eight items at the trial level (*N* = 1,278). Sampling adequacy was excellent, KMO = 0.955, and Bartlett’s test of sphericity was significant, χ^2^(28) = 11062.835, *p* < 0.001. A single component with an eigenvalue greater than 1 emerged (eigenvalue = 6.376), accounting for 79.697% of the total variance; the second eigenvalue dropped sharply to 0.376, and the scree plot showed a clear one-factor solution. All eight items loaded strongly on this single component (loadings 0.878–0.909). These results indicate that, in the present data, the eight items measure one general dimension—plausibly a generalized positive evaluation of the excerpt—rather than four separable constructs. Accordingly, the four content domains proposed *a priori* are not treated as distinct dimensions in this study: the overall aesthetic evaluation score serves as the sole outcome variable in hypothesis testing, and the domain-level results in Section 5.6.5 are presented as exploratory only. The very high α coefficients reported above should likewise be read in light of this redundancy, as semantically overlapping items inflate internal-consistency estimates without demonstrating structural validity.

### 5.5 Testing H1: relationship between actual compositional condition and creator attribution judgment

#### 5.5.1 Confusion matrix and attribution tendencies

The distribution of listeners’ creator attribution judgments across the three actual compositional conditions is presented in Table 7. In the AI-generated and human–AI collaborative conditions, the proportions of the three attribution categories were close to a uniform distribution. In contrast, in the human-composed condition, the proportion of excerpts attributed to human composition was clearly concentrated, reaching 45.3%. This indicates a distinct attribution tendency toward the human-composed category.

**Table 7:** Confusion matrix between actual compositional condition and creator attribution judgment (*N* = 1,278).

Actual compositional condition	Attributed to AI generation *n* (%)	Attributed to human-AI collaboration *n* (%)	Attributed to human composition *n* (%)	Total *n* (%)
AI-generated	133 (31.2%)	155 (36.4%)	138 (32.4%)	426 (100%)
Human-AI collaborative composition	138 (32.4%)	156 (36.6%)	132 (31.0%)	426 (100%)
Human-composed	106 (24.9%)	127 (29.8%)	193 (45.3%)*	426 (100%)
Total	377 (29.5%)	438 (34.3%)	463 (36.2%)	1,278 (100%)

Values in parentheses are row percentages within each actual compositional condition. Chi-square test: χ^2^(4) = 23.076, *p* < 0.001; Phi = 0.134; Cramer’s V = 0.095. * In the human-composed condition, the proportion attributed to human composition (45.3%) was significantly higher than the uniform-distribution baseline (33.3%).

#### 5.5.2 Chi-square test results

The results of the Pearson chi-square test are also presented in Table 7. A statistically significant association was found between actual compositional condition and creator attribution judgment, χ^2^(4) = 23.076, *p* < 0.001. However, the effect size was very small, Cramér’s V = 0.095, indicating a weak association. With 1,278 observations, even near-null effects readily reach statistical significance; the significance of the chi-square test should therefore not be read as evidence of a substantively strong relationship between actual source and attribution. All expected cell frequencies were greater than 5, indicating that the assumptions for the chi-square test were satisfied.

#### 5.5.3 Conclusion

The above results indicate that H1 was partially supported. A significant association was observed between actual compositional condition and creator attribution judgment. However, this association was very weak (Cramér’s V = 0.095) and was mainly driven by the attribution tendency in the human-composed condition. Even there, only 45.3% of genuinely human-composed excerpts were attributed to human composition, against a chance baseline of 33.3%; that is, the majority (54.7%) of human-composed excerpts were misattributed to AI generation or to human–AI collaboration. Attribution judgments in the AI-generated and human–AI collaborative conditions were essentially at chance. Taken together, these results indicate that listeners with formal music training could not reliably identify the compositional source of the excerpts, with only a modest advantage for recognizing human composition. This near-null discriminability is treated as a substantive finding in its own right in Section 6.1.

#### 5.5.4 Supplementary analysis of attribution confidence

The results of the one-way repeated-measures ANOVA with attribution confidence as the dependent variable are presented in Table 8. The assumption of sphericity was met, W = 0.996, *p* = 0.870. Actual compositional condition had a significant effect on attribution confidence, F(2, 140) = 8.043, *p* < 0.001, partial η^2^ = 0.103.

**Table 8:** Repeated-measures ANOVA of attribution confidence across compositional conditions (*N* = 71).

Compositional condition	M	SD	ANOVA	Bonferroni comparisons (*p*)	Partial η^2^
AI-generated	4.524	0.976	F(2, 140) = 8.043, *p* < 0.001	AI vs. Human-AI: ns AI vs. Human: *p* = 0.002 Human-AI vs. Human: *p* = 0.004	0.103
Human-AI collaborative composition	4.540	0.882			
Human-composed	4.754	1.025			

Confidence was measured on a 7-point scale (1 = completely uncertain, 7 = highly certain). ns = not significant (*p* = 1.000).

Bonferroni-corrected *post hoc* comparisons showed that confidence in the human-composed condition was significantly higher than that in the AI-generated condition, *p* = 0.002, and the human–AI collaborative condition, *p* = 0.004. No significant difference was found between the AI-generated and human–AI collaborative conditions, *p* = 1.000.

Taken together with the chi-square results for H1, these findings suggest that listeners were not only more likely to attribute human-composed materials to human creation, but also showed relatively higher subjective confidence in these judgments.

### 5.6 Testing H2: main effect of actual compositional condition on aesthetic evaluation

#### 5.6.1 Main effect and post hoc comparisons

The results of the one-way repeated-measures ANOVA with the overall aesthetic evaluation score as the dependent variable are presented in Table 9. The assumption of sphericity was met, W = 0.979, *p* = 0.483. The main effect of actual compositional condition was significant, F(2, 140) = 21.252, *p* < 0.001, partial η^2^ = 0.233, observed power = 1.000.

**Table 9:** One-way repeated-measures ANOVA of aesthetic evaluation across compositional conditions (*N* = 71).

Content domain	AI-generated M (SD)	Human-AI collaborative M (SD)	Human-composed M (SD)	F(2, 140)	*p*	Partial η^2^
Overall aesthetic evaluation	3.823 (0.981)	4.007 (0.955)	4.252 (1.002)	21.252	< 0.001	0.233
Liking	3.843 (1.028)	4.045 (0.990)	4.295 (1.053)	18.681	< 0.001	0.211
Beauty	3.805 (1.004)	3.975 (0.957)	4.230 (1.019)	17.528	< 0.001	0.200
Perceived musical quality	3.930 (0.978)	4.088 (0.952)	4.354 (1.012)	21.076	< 0.001	0.231
Emotional expressiveness	3.716 (1.038)	3.919 (1.055)	4.127 (1.041)	15.791	< 0.001	0.184

After Bonferroni correction, all pairwise comparisons among the three conditions were significant (all *p* ≤ 0.05). In the beauty domain, the difference between AI-generated and human-AI collaborative composition reached the threshold of statistical significance (*p* = 0.050). Domain-level results are exploratory; see Section 5.4 for evidence of a single-factor structure.

Bonferroni-corrected pairwise comparisons showed that, within the range of materials used in this study, all three compositional conditions differed significantly from one another. The results followed a graded pattern of human-composed > human–AI collaborative > AI-generated.

#### 5.6.2 Conclusion

Under blind-listening conditions, actual compositional condition had a significant effect on listeners’ aesthetic evaluations. Therefore, H2 was supported. However, this result should be interpreted with caution, given the possible residual differences in material quality.

#### 5.6.3 Exploratory analysis of the four item domains

The repeated-measures ANOVA results for the four content domains are also presented in Table 9. Significant main effects were found for liking, beauty, perceived musical quality, and emotional expressiveness, all *p* < 0.001. The mean-score ordering was consistent with that of the overall aesthetic evaluation score.

In the beauty domain, the difference between the AI-generated and human–AI collaborative conditions reached the threshold of statistical significance, *p* = 0.050. All other pairwise comparisons reached higher levels of statistical significance. Given the single-factor structure reported in Section 5.4, these domain-level analyses are exploratory and should not be interpreted as evidence of four separable constructs. The near-identical pattern and magnitude of effects across the four domains is, in fact, consistent with the conclusion that they index a single underlying evaluative dimension.

### 5.7 Testing H3: independent association between creator attribution judgment and aesthetic evaluation

#### 5.7.1 Model specification and fixed effects

The results of the linear mixed-effects model are presented in Table 10, estimated using REML, AIC = 3354.812. The fixed effects of creator attribution judgment, F(2, 1225.4) = 89.118, *p* < 0.001, actual compositional condition, F(2, 1202.4) = 11.458, *p* < 0.001, and attribution confidence, F(1, 1271.9) = 206.376, *p* < 0.001, were all statistically significant.

**Table 10:** Linear mixed-effects model predicting aesthetic evaluation from creator attribution judgment (*N* = 1,278).

Predictor	B	SE	t	df	*p*	95% CI
Creator attribution judgment (reference: attributed to human composition)						
Attributed to AI generation	−0.808	0.061	−13.213	1219.4	< 0.001	[−0.928, −0.688]
Attributed to human–AI collaboration	−0.501	0.062	−8.145	1233.7	< 0.001	[−0.622, −0.381]
Actual compositional condition (reference: human-composed)						
AI-generated condition	−0.264	0.057	−4.633	1202.9	< 0.001	[−0.376, −0.152]
Human–AI collaborative condition	−0.076	0.057	−1.330	1202.8	0.184	[−0.188, 0.036]
Covariate						
Attribution confidence	0.346	0.024	14.366	1271.9	< 0.001	[0.299, 0.393]
Estimated marginal means (confidence fixed at M = 4.606)						
Attributed to human composition (reference)	4.437	0.101	–	88.0	–	[4.238, 4.637]
Attributed to human–AI collaboration	3.936	0.101	–	89.7	–	[3.735, 4.137]
Attributed to AI generation	3.629	0.102	–	92.8	–	[3.427, 3.832]

REML estimation; degrees of freedom approximated by the Satterthwaite method; AIC = 3354.812, BIC = 3365.108. Participant-level random-intercept variance = 0.600, SE = 0.108, Wald Z = 5.546, *p* < 0.001. For estimated marginal means (EMMs), df refers to the Satterthwaite denominator degrees of freedom of the estimate. After Bonferroni correction, all pairwise comparisons among the three EMMs were significant (all *p* < 0.001).

Using trials attributed to human composition as the reference category, trials attributed to AI generation received significantly lower aesthetic scores, B = −0.808, *p* < 0.001. Trials attributed to human–AI collaborative composition also received significantly lower aesthetic scores, B = −0.501, *p* < 0.001. When attribution confidence was fixed at its mean value, 4.606, the estimated marginal mean was highest for trials attributed to human composition, M = 4.437, followed by trials attributed to human–AI collaborative composition, M = 3.936, and trials attributed to AI generation, M = 3.629. All pairwise comparisons among the three attribution categories were significant, all *p* < 0.001.

In addition, after controlling for creator attribution judgment, the effect of actual compositional condition was mainly reflected in the difference between the AI-generated and human-composed conditions, B = −0.264, *p* < 0.001. The difference between the human–AI collaborative and human-composed conditions was no longer significant, B = −0.076, *p* = 0.184.

#### 5.7.2 Random effects

The random-effects results showed that the estimated variance of the participant-level random intercept was 0.600, SE = 0.108, Wald Z = 5.546, *p* < 0.001. The model fit indices were AIC = 3354.812 and BIC = 3365.108. These results confirm that significant individual differences existed in participants’ overall rating tendencies, supporting the necessity of including participant-level random intercepts in the model.

#### 5.7.3 Sensitivity analysis excluding attribution confidence

Because attribution confidence is plausibly downstream of the same process that generates both attribution judgments and aesthetic ratings, its inclusion as a covariate may constitute adjustment for a mediator. To assess the sensitivity of the attribution effect to this modeling decision, the mixed-effects model was re-estimated without the confidence covariate, retaining creator attribution judgment and actual compositional condition as fixed effects and the participant-level random intercept.

The attribution effect was robust to this change and, if anything, larger. Creator attribution judgment remained highly significant, F(2, 1222.6) = 104.221, *p* < 0.001. Trials attributed to AI generation received lower aesthetic ratings than trials attributed to human composition, B = −0.928, SE = 0.065, *p* < 0.001, 95% CI [−1.055, −0.800], compared with B = −0.808 in the primary model; the corresponding estimate for trials attributed to human–AI collaboration was B = −0.583, SE = 0.066, *p* < 0.001, 95% CI [−0.712, −0.454], compared with B = −0.501. Estimated marginal means were 3.573, 3.918, and 4.501 for trials attributed to AI generation, human–AI collaboration, and human composition, respectively, with all Bonferroni-corrected pairwise comparisons significant (all *p* < 0.001). Thus, the H3 conclusion does not depend on the inclusion of the confidence covariate; the modest attenuation of the attribution coefficients when confidence is included is consistent with confidence carrying part of the shared variance, as would be expected if it is a downstream correlate of the same evaluative process.

One difference between the two specifications should be noted. Without the confidence covariate, the fixed effect of actual compositional condition showed a significant difference between the human–AI collaborative and human-composed conditions, B = −0.135, *p* = 0.027, whereas this contrast was not significant in the primary model, *p* = 0.184. The claim that this condition difference is fully accounted for by attribution judgment therefore holds only when confidence is also controlled, and is interpreted with corresponding caution in Section 6.3.

#### 5.7.4 Within-condition analyses

Because attribution judgments and aesthetic ratings were collected within the same trial, the association observed in the full model is, in principle, open to a reverse-inference interpretation, in which liking drives attribution rather than the reverse, or to a common-cause interpretation, in which some third property of the excerpt drives both. As a partial test of these alternatives, the mixed-effects model was re-estimated separately within each actual compositional condition (*n* = 426 per condition), with creator attribution judgment as a fixed factor, attribution confidence as a covariate, and a participant-level random intercept. Within each condition, the actual source of the excerpts is held constant by construction, so any association between attribution and aesthetic rating cannot reflect true source differences.

The attribution effect remained significant within all three conditions. Critically, within the AI-generated condition, where every excerpt shared the same true source, creator attribution judgment still showed a significant effect on aesthetic evaluation, F(2, 384.6) = 31.874, *p* < 0.001. Trials attributed to AI generation received lower ratings than trials attributed to human composition, B = −0.868, SE = 0.109, *p* < 0.001, as did trials attributed to human–AI collaboration, B = −0.499, SE = 0.110, *p* < 0.001; estimated marginal means were 3.408, 3.777, and 4.276 for trials attributed to AI generation, human–AI collaboration, and human composition, respectively, and all Bonferroni-corrected pairwise comparisons were significant (all *p* ≤ 0.002). The same pattern held within the human–AI collaborative condition, F(2, 385.6) = 15.093, *p* < 0.001 (attributed-AI vs. attributed-human, B = −0.630, *p* < 0.001), and within the human-composed condition, F(2, 393.0) = 22.627, *p* < 0.001 (attributed-AI vs. attributed-human, B = −0.703, *p* < 0.001), although in these two conditions the difference between trials attributed to AI generation and those attributed to human–AI collaboration did not reach significance (*p* = 0.136 and *p* = 0.484, respectively).

These within-condition results, particularly the robust attribution effect within the AI-generated condition, indicate that the association between attribution judgment and aesthetic evaluation is not reducible to genuine quality differences between compositional conditions. They do not, however, establish the direction of the association, which remains an open question for designs employing label manipulation (see Section 7).

#### 5.7.5 Crossed random-effects robustness check

The primary model included a random intercept for participants only. Because repeated ratings of the same 18 stimuli violate the independence assumption at the item level, a crossed random-effects model was estimated as a robustness check, with random intercepts for both participant and stimulus and the same fixed effects as the primary model (creator attribution judgment, actual compositional condition, and attribution confidence), using REML estimation with Satterthwaite degrees of freedom.

The stimulus-level random-intercept variance was estimated at the boundary of the parameter space (variance = 0), and SPSS accordingly flagged the parameter as redundant and issued a non-positive-definite Hessian warning; this warning is the expected consequence of a boundary estimate rather than an indication of model misspecification. Substantively, the boundary estimate indicates that, once the fixed effects were accounted for, no additional between-stimulus variance in aesthetic ratings was detectable in these data. As a result, the crossed model reduced to the participant-only model, and all fixed-effect estimates were identical to those of the primary model to three decimal places: creator attribution judgment, F(2, 1225.4) = 89.118, *p* < 0.001, with attributed-AI vs. attributed-human B = −0.808 and attributed-collaboration vs. attributed-human B = −0.501; actual compositional condition, F(2, 1202.4) = 11.458, *p* < 0.001; and attribution confidence, B = 0.346, *p* < 0.001.

The H3 attribution effect is therefore fully robust to the inclusion of stimulus-level random intercepts. Two qualifications apply. First, with only 18 stimuli, six per condition, the stimulus-level variance is estimated with little information, and a boundary estimate of zero should not be interpreted as demonstrating the absence of stimulus heterogeneity. Second, because each stimulus is nested within one compositional condition, the fixed effect of condition absorbs between-condition stimulus differences by construction; the structural coupling between stimulus and condition therefore remains a limitation of the design (see Section 7).

#### 5.7.6 Conclusion

After simultaneously controlling for actual compositional condition, attribution confidence, and individual participant differences, listeners’ creator attribution judgments remained significantly and independently associated with aesthetic evaluation. Therefore, H3 was supported. Because attribution and aesthetic rating were measured simultaneously within each trial, this association is interpreted as associational rather than causal. This finding indicates that, under blind-listening conditions, listeners’ spontaneously formed creator attribution judgments were independently associated with aesthetic evaluation, and that this attribution effect existed independently of the actual compositional condition of the musical excerpts.

## 6 Discussion and conclusion

### 6.1 Trained listeners cannot reliably identify AI-generated music: a weak human-composition advantage

The most striking aspect of the H1 results is not the significant association itself, which was very small (Cramér’s V = 0.095), but what it reveals: listeners with three or more years of conservatory-level training could not reliably identify AI-generated music produced by a small-corpus personal-style generator. Attribution judgments in the AI-generated condition were essentially at chance, and even in the human-composed condition the majority of excerpts were misattributed. This near-null discriminability is a substantive finding in its own right. Against this baseline of general indistinguishability, only one systematic tendency emerged: a modest concentration of human attributions (45.3% versus a 33.3% chance baseline) for genuinely human-composed materials. By contrast, attribution judgments for the AI-generated and human–AI collaborative conditions were both close to a random distribution, and the judgment patterns of these two conditions were highly similar and difficult to distinguish. This pattern is consistent with Shank et al.’s (2023) finding that listeners may hold biases toward AI-created music. It also suggests that human–AI collaborative composition has not yet established an independently identifiable perceptual identity at the level of listener judgment.

Notably, although the human–AI collaborative works were modified by the composer to a limited extent, their attribution-judgment distribution remained highly similar to that of the AI-generated works. This suggests that listeners did not form a clear tendency to distinguish between these two types of materials. The result points to a possible asymmetry between composer intervention, aesthetic evaluation, and perceived creative identity. In H2, human–AI collaborative works received higher aesthetic ratings than AI-generated works, yet in H1 they did not acquire a perceptual identity clearly distinct from AI-generated works. This finding provides an important reference for understanding the perceptual boundaries of human–AI collaborative composition.

### 6.2 Graded effect of actual compositional condition on aesthetic evaluation

The support for H2 indicates that, under blind-listening conditions, aesthetic evaluations of the three actual compositional conditions showed a directional and cross-domain consistency. Overall, the results followed a graded pattern of human-composed > human–AI collaborative > AI-generated. This pattern was observed across the four content domains of liking, beauty, perceived musical quality, and emotional expressiveness.

This finding is consistent with the direction of the human-creation advantage observed by Bellaiche et al. (2023) and Tubadji et al. (2021) under external-label conditions. However, the key difference in the present study is that the aesthetic differences occurred under blind-listening conditions, without source information being provided, rather than being driven by identity labels imposed by the researchers. This suggests that even in the absence of explicit authorship cues, the actual compositional condition of the work may still be systematically associated with listeners’ aesthetic evaluations through integrated auditory differences at the material level.

In addition, human–AI collaborative works received higher scores than purely AI-generated works across the content domains, suggesting that composer intervention may be associated with more positive aesthetic evaluation. Nevertheless, although this study attempted to control stimulus differences through expert screening, unified sound sources, identical export parameters, similar duration, tempo range, register, and rhythmic density, the three groups of materials were ultimately produced through different creative processes. Therefore, actual compositional condition and individual material quality may still be partially intertwined. The aesthetic gradient revealed by H2 should not be interpreted as a pure causal effect of compositional condition on aesthetic evaluation. Rather, it should be understood as a perceptual difference observed under the material-control conditions of the present study.

### 6.3 Independent association between attribution judgment and aesthetic evaluation

Compared with H1 and H2, H3 more directly addresses the central psychological question of this study: whether listeners’ aesthetic evaluations are related not only to the actual source of the work, but also to their subjective attribution judgments. After controlling for actual compositional condition and individual participant differences, creator attribution judgment remained significantly and independently associated with aesthetic evaluation. Trials attributed to human composition consistently received the highest aesthetic scores, whereas trials attributed to AI generation received the lowest scores. This indicates that aesthetic evaluation is associated not only with the actual source of the work, but also with listeners’ subjective attribution of that source.

It must be emphasized that the present design cannot establish the direction of this association. Because attribution judgments and aesthetic ratings were collected within the same trial, at least three accounts are equally consistent with the data: attribution may shape evaluation; evaluation may drive attribution, such that listeners who like an excerpt infer that a human composed it; or a third property of the excerpt, such as melodic coherence or cadential conviction, may independently drive both. The within-condition analyses reported in Section 5.7.4 show that the association persists when the actual source is held constant, which rules out true source differences as a sufficient explanation, but they cannot adjudicate between the attribution-to-evaluation and evaluation-to-attribution readings. The term “endogenous perceptual bias” is therefore used here to denote a stable association between spontaneous attribution and aesthetic evaluation, not a demonstrated causal mechanism. Label-manipulation designs, in which attribution is experimentally assigned while materials are held constant, are required to establish direction (see Section 7).

A further finding concerns the difference in aesthetic evaluation between the human–AI collaborative and human-composed conditions. In the primary model, which controlled for both attribution judgment and attribution confidence, this difference was no longer significant (*p* = 0.184); in the sensitivity model without the confidence covariate, it remained significant but was substantially attenuated (B = −0.135, *p* = 0.027) relative to the raw difference observed in H2. This pattern suggests that a considerable part of the aesthetic gap between human–AI collaborative and human-composed works is associated with listeners’ attribution judgments rather than reflecting only direct differences in the musical materials, although the strength of this conclusion depends partly on the treatment of the confidence covariate.

Taken together, the evidence suggests that the observed effects reflect both sources rather than either alone. Genuine material differences contribute: actual compositional condition retained a significant effect on aesthetic evaluation even after attribution was controlled (Section 5.7.1), indicating that the AI-generated excerpts differed from the human-composed excerpts in ways listeners responded to regardless of their attributions. At the same time, attribution-linked variance is not reducible to material quality: the attribution–evaluation association persisted within each compositional condition, including within the AI-generated condition where the true source of all excerpts was identical (Section 5.7.4). The two contributions are therefore distinguishable in the data, although, as noted above, the direction of the attribution-linked component cannot be established with the present design.

Previous studies have shown that external labels imposed by researchers can alter aesthetic judgments (Bellaiche et al., 2023; Shank et al., 2023). The present study further shows that even without external labels, listeners’ spontaneously formed creator attribution judgments are also stably associated with aesthetic evaluation. This result provides preliminary empirical evidence for endogenous perceptual bias as a potential psychological mechanism in the reception of AI-generated music.

### 6.4 Conclusion

This study systematically examined the construction process and perceptual reception of a personal compositional style model under human–AI collaborative conditions from both practice-based and empirical perspectives. At the level of practice, the study documented how a composer without programming expertise gradually transformed personal melodic style into executable generative rules through sustained natural-language interaction. This process, conceptualized as “perceptual rule transformation,” provides an analyzable and transferable practical example for human–AI collaborative modeling.

At the empirical level, the results of the blind-listening experiment generally supported the hypotheses of this study, although the degree of support varied. H1 was partially supported: the association between actual source and attribution was statistically significant but very small, and its most notable implication is that trained listeners could not reliably identify AI-generated music. H2 showed that aesthetic evaluations differed across the three types of materials, although this finding should be interpreted cautiously. H3 constituted the most central psychological finding of the study.

Taken together, these findings suggest that the aesthetic reception of AI-generated music is not merely a direct perception of musical material, but also a subjective evaluative process related to listeners’ creator attribution judgments. Listeners’ creator attribution judgments are stably associated with aesthetic evaluation, and together with the actual source of the work, they constitute important factors in aesthetic judgment. This conclusion contributes to the evaluation of the reception of personal-style generative systems, helps clarify the perceptual logic of human–AI collaborative composition, and provides a reference for further discussion of the relationship between creative-agent identity and aesthetic evaluation.

## 7 Limitations and future directions

Several limitations of this study should be considered when interpreting the findings. First, all participants were music-major students with systematic listening and aural-training backgrounds. Their patterns of creator attribution judgment and aesthetic evaluation may not represent those of non-professional listeners. The external validity of the findings therefore requires further examination across different listener groups.

Second, each compositional condition included only six stimulus excerpts. Individual material differences and actual compositional condition were therefore partially overlapping, which may have influenced the results of attribution judgment and aesthetic evaluation. Although this study attempted to reduce material-quality differences through expert screening and unified audio-production standards, it could not completely eliminate the structural coupling between material-level differences and actual compositional condition. Although the crossed random-effects robustness check (Section 5.7.5) showed that the key results were unchanged when a stimulus-level random intercept was added, the small number of stimuli means that item-level variance was estimated with limited precision, and the reported significance levels should be interpreted with this constraint in mind.

Third, a distinct limitation concerns the multiple roles played by the first author, who simultaneously served as the source of the training corpus, the director of the generator’s construction, the producer of the AI-generated stimuli, the reviser in the human–AI collaborative condition, and the composer of the human-composed condition. The human-composed condition therefore does not represent human composition in general, but the output of one composer. Because the human-composed excerpts were pre-existing works written between 2020 and 2023, before the generator was constructed, demand awareness at the time of composition can be excluded; however, the possibility remains that the selection of these works as stimuli was implicitly shaped by the author’s expectations. The three-condition design of the present study cannot fully distinguish these possibilities, and replication with independent composers and independently sourced human materials is required.

Fourth, the training corpus consisted only of sixteen vocal works by the researcher, representing an extremely small-sample learning context within a single personal style. Whether this methodological pathway can be applied to other styles or composer groups remains to be verified.

Fifth, this study did not systematically collect background variables such as gender, academic year, and years of music study, which limited further analysis of the sources of individual differences among participants.

Sixth, although the aesthetic evaluation scale showed very high internal consistency, principal component analysis indicated a single-factor structure, and the paired items within each content domain were semantically similar. The high reliability coefficients therefore partly reflect item redundancy rather than measurement precision, and the scale in its present form cannot distinguish liking, beauty, perceived quality, and emotional expressiveness as separate constructs. Future studies should employ items designed to maximize discriminant validity across these domains.

Future research may be extended in several directions. First, non-music-major listeners could be included to compare the influence of professional training on endogenous perceptual bias. Second, different degrees of human–AI collaborative intervention could be introduced to examine the relationship between the amount of composer involvement and perceptual identifiability. Third, the present paradigm could be replicated using personal corpora from more composers to test the cross-style applicability of the “perceptual rule transformation” method. Fourth, experiments using homogeneous AI-generated materials or label manipulations could be introduced to examine whether subjective attribution still predicts aesthetic evaluation when the actual source is held constant, thereby further excluding the competing explanation of material-quality differences. Fifth, longitudinal designs could be adopted to track changes in listeners’ attribution judgments and aesthetic evaluations after repeated exposure to human–AI collaborative music.

## Data Availability

The datasets, stimulus audio files and analysis code generated for this study can be found in the Open Science Framework (OSF) repository: https://doi.org/10.17605/OSF.IO/ZUSJN.
